# A bridge between trust and control: computational workflows meet automated battery cycling†

**DOI:** 10.1039/d3ta06889g

**Published:** 2024-04-03

**Authors:** Peter Kraus, Edan Bainglass, Francisco F. Ramirez, Enea Svaluto-Ferro, Loris Ercole, Benjamin Kunz, Sebastiaan P. Huber, Nukorn Plainpan, Nicola Marzari, Corsin Battaglia, Giovanni Pizzi

**Affiliations:** a Materials for Energy Conversion, Empa Überlandstr. 129 8600 Dübendorf Switzerland corsin.battaglia@empa.ch; b Laboratory for Materials Simulations (LMS), National Centre for Computational Design and Discovery of Novel Materials (MARVEL), Paul Scherrer Institute 5232 Villigen Switzerland giovanni.pizzi@psi.ch; c Theory and Simulations of Materials (THEOS), National Centre for Computational Design and Discovery of Novel Materials (MARVEL), École Polytechnique Fédérale de Lausanne 1015 Lausanne Switzerland; d ETH Zurich, Department of Information Technology and Electrical Engineering Gloriastrasse 35 8092 Zurich Switzerland; e Institute of Materials (IMX), École Polytechnique Fédérale de Lausanne 1015 Lausanne Switzerland; f Technische Universität Berlin, Centre for Advanced Ceramic Materials Hardenbergstr. 40 10623 Berlin Germany

## Abstract

Compliance with good research data management practices means trust in the integrity of the data, and it is achievable by full control of the data gathering process. In this work, we demonstrate tooling which bridges these two aspects, and illustrate its use in a case study of automated battery cycling. We successfully interface off-the-shelf battery cycling hardware with the computational workflow management software AiiDA, allowing us to control experiments, while ensuring trust in the data by tracking its provenance. We design user interfaces compatible with this tooling, which span the inventory, experiment design, and result analysis stages. Other features, including monitoring of workflows and import of externally generated and legacy data are also implemented. Finally, the full software stack required for this work is made available in a set of open-source packages.

## Abstract 2

We demonstrate a link between workflow management and instrument automation tools, effectively bridging “trust” from tracking data provenance with automated “control” of experiments. We illustrate our approach using a battery cycling case study.

## Graphical abstract

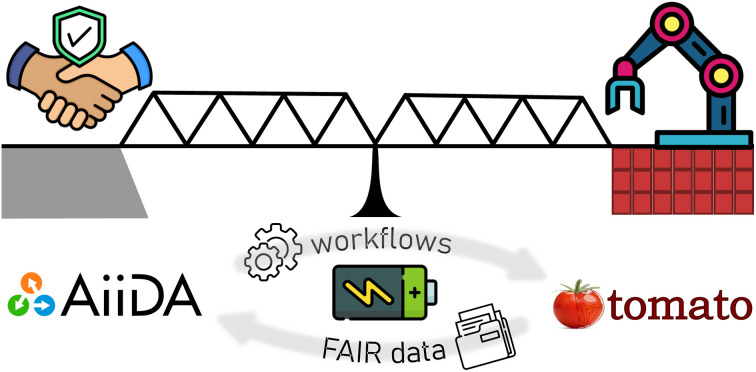

## 1 Introduction

“Trust is good, control is better”. This apocryphal quote, often attributed to Lenin, might be interpreted as a guiding principle for good research data management (RDM) practices. The “trust” aspect of RDM is codified in guidelines for RDM,^1^ and implemented by overarching standards^2^ as well as grassroots approaches, such as small data.^3^ However, the “control” aspect of RDM has a large rift between the computational and experimental domains. Researchers in computational materials science tend to focus on implementing code interoperability^4^ and developing workflow management tools.^5–8^ On the other hand, experimental researchers are (still) tackling digitalisation, mainly *via* electronic lab notebooks^9,10^ and instrument automation.^11–13^

Meanwhile, it is clear that data management plans should be designed to cover all data generated in cross-disciplinary projects,^14^ by treating experimental as well as computational data on an even footing from an RDM point of view. Such integrated solutions could then build on the advantages gained from implementing FAIR data principles,^2^ for instance enabling automated materials discovery using existing tools,^5,13^ often interfaced with machine learning models^15^ in a distributed fashion.^16^ Indeed, several frameworks capable of storing and distributing such integrated data are being built.^17–19^

However, a bridge between “trust” and “control”, compatible with both computational and experimental data, yet targetting off-the-shelf instrumentation and reusing common software tools, currently does not exist. For instance, the ARChemist project focuses on sample synthesis^20^ and sample preparation,^21^ and while its powder-bot used for X-ray diffraction analysis is interfaced with common diffractometers,^22^ it is not driven as part of a computational workflow. However, in the A-Lab project, automated X-ray diffraction driven by machine learning algorithms has been demonstrated,^23^ driven by bespoke, but open-source software. Similarly, in the Matter Lab, large language model-driven synthesis and wet chemistry has been successfully demonstrated.^24^ However, the orchestration of such tasks remains “tailored to specific setups or [is not yet] implemented for real-world synthesis”.^25^ It also relies on the use of custom orchestrators.

In order to increase adoption and interoperability of RDM practices, the use of common, established, open-source orchestrators or workflow manager softwares (WFMSs) is, in our view, crucial. In a previous work, Stricker *et al.* demonstrated a proof-of-concept control of a bespoke experimental set-up^26^ using an established computational WFMS pyiron.^7^ In this work, we present how our autonomous robotic battery materials research platform, Aurora,^27^ has been integrated with the WFMS AiiDA,^8,28^ originally designed for computational science.^29^ In contrast with the previous works discussed above, here a WFMS is taught to interact with an off-the-shelf potentiostat using a shim around vendor-supplied libraries, exploiting existing plugin architectures. Such integration enables the coupling of experiments with computational workflows using digital twins. The ultimate goal for the Aurora platform, developed as part of the Battery Interface Genome-Materials Acceleration Platform (BIG-MAP),^30^ is to link physics- and data-based modelling with automated orchestration of battery assembly and battery cycling experiments, forming a closed-loop sequence. Additionally, we discuss the development of user interfaces that are familiar to experimentalists but compatible with computational tools, implemented with the graphical web platform AiiDAlab,^31^ based on AiiDA. Finally, we discuss new features incorporated into AiiDA, that allow for an automated supervision of experimental (and computational) tasks during their runtime. In the following Implementation section, we first outline the project components and their design philosophy. In particular, we focus on the elements of our software stack developed as part of this work. In the Results section, we present a case study using a range of coin cell batteries built by an automated coin cell assembly robot, illustrating the user experience and outlining the applications of our work.

## 2 Implementation

**Fig. 1:**
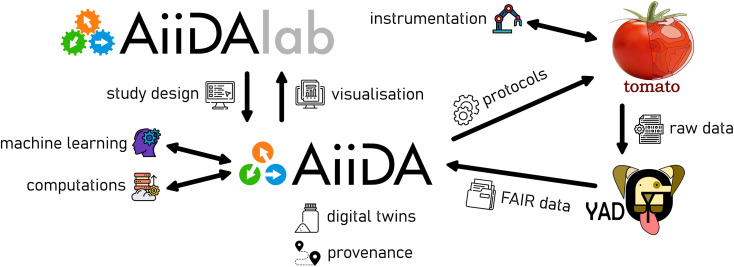
Overview of the software stack used in the current work, and the key concepts and processes. All software shown in the above diagram is available *via* the BIG-MAP app store at https://big-map.github.io/big-map-registry/, under an open-source license.

An overview of the software components, which create our bridge between trust and control, is shown in Fig. 1. The central pillar is formed by AiiDA,^8^ providing a base of trust by tracking the provenance of data, collating it on a per-sample basis into digital twins, which are further discussed below. In this implementation, the human-facing aspect of control is provided by AiiDAlab,^31^ allowing users to design their studies *via* a web-based user interface, which can be rapidly prototyped and easily deployed using modular components. Data visualisation and reporting can be implemented *via* AiiDAlab. Examples of user interfaces, developed for battery materials research and packaged into the AiiDA-Aurora plugin, are presented in the Case study section, below. Automated control *via* external modules, using *e.g.* machine learning approaches, as well as AiiDA's interface for computational tasks, are core and well established features of the AiiDA ecosystem, and are discussed elsewhere.^8^

### 2.1 Tomato: automation without the pain!

The link to the experimental instrumentation is provided by interfacing AiiDA with our instrument automation platform, tomato, developed for this purpose. The Python-based tomato includes a job scheduling daemon, device drivers, and a user front-end utility called ketchup. The usage patterns of this library are modelled after common scheduling tools, such as Sun Grid Engine, OpenPBS or Torque, familiar to users of high-performance computing. We refer the keen reader to the online project documentation for further details.‡‡See the usage section of the tomato documentation at: https://dgbowl.github.io/tomato/master/usage.html However, other instrument automation platforms could be interfaced with AiiDA by writing an appropriate scheduler plugin.

Within tomato, the user supplies a definition of *devices*, which represent and track the state of the controlled instruments. Such *devices* can be arbitrarily combined and addressed into *pipelines*. For instance, in a multi-channel potentiostat, each channel of the *device* can be addressed independently using the appropriate *pipeline*. The experimental protocols (YAML or JSON files) can be either written and submitted manually, or deployed by AiiDA. The protocols are interpreted into a language understood by the hardware by tomato's drivers. This implies the development of ontologies, or vendor-agnostic domain specific languages; see *e.g.* ref. 32 and 33 for current efforts in heterogeneous catalysis and in the battery communities, respectively. The parsing of the raw instrumental data into a FAIR data format, understood and stored in AiiDA, is performed automatically and periodically by the FAIR data parser yadg.^34^ Currently, tomato supports a wide range of BioLogic potentiostats, using a Python-based shim around a vendor-supplied DLL. An extension of the driver library is currently underway.

### 2.2 Towards digital twins

One key conceptual difference between the computational and experimental domains concerns the objects under study, *i.e.* the “samples”. In the computational domain, a sample is an immutable concept: a set of atomic coordinates forming an input of an electronic structure calculation, or a set of boundary conditions for a combustion problem. The outcome of the calculation is usually a property of the original sample (*e.g.* the energy of that structure, or the adiabatic flame temperature for that mixture and state); sometimes, a new sample may be generated by the calculation (*e.g.* a relaxed geometry, or an outlet mixture composition and its state). Each of these samples can be easily and separately represented by a digital twin, and each of those samples can be reused in further computations, if necessary.

In experimental research, this ideal situation is complicated by sample history. For samples representing a single object (*e.g.* a coin cell battery), an experiment irreversibly changes the sample, so that the sample corresponding to the original state is no longer available for new experiments; only the sample corresponding to the current state is. Formally, it is simpler to consider this state-change to be always present, even for so-called “non-destructive” testing methods. Approaches for extending the digital twins and provenance graphs to allow for such bookkeeping within AiiDA are being trialled. However, in the current work, the unmodified AiiDA provenance model is used, with sample history tracked using timestamps.

Another complication arises from the practicalities of experimental lab work: the state of the sample might be modified by user intervention outside of the scope of an AiiDA workflow. The current gold standard in lab practice is to track all activities in the lab in a lab book (supplemented by a lab inventory management system), which then contains the full authoritative sample history. An additional difference with respect to the computational domain is that storage of experimental samples can also affect the sample state. In the case of reactive materials used in battery research, keeping track of storage parameters such as the composition of the atmosphere and duration of storage is part of best practice.^35^ Therefore, interfaces between electronic lab notebooks and WFMSs, and their wider adoption, are necessary to fully address this issue; such an interface will be discussed in a further work.

Finally, it can be argued that the necessary features of a digital twin include (i) a multi-physics or data driven model of an object, accompanied by (ii) real-world data related to the object.^36^ Indeed, in the NASA definition of digital twins, the modelling forms the backbone into which the measured (“sensor”) data is integrated.^37^ Furthermore, the model should be able to (iii) self-adjust using the measured data in a closed-loop sequence.^36^ In the current work, we focus on the integration of data from automated experiments into AiiDA's provenance, *i.e.* the second of the three requirements in the above definition of a digital twin. As for the other two requirements, as shown in Fig. 1, AiiDA already allows for closing the loop by integration of machine learning models with methods used in computational chemistry (*e.g.* in metallurgy^38^ or catalysis^39^). Similarly, the ability to carry out computational tasks based on experimental data attached to an AiiDA digital twin has been recently demonstrated^40^ using the AiiDAlab web platform.

### 2.3 Automated task supervision

Computational tasks in a workflow are generally considered as atomic transactions: the WFMS submits the task to the calculator *via* a scheduling software, which then reports back to the WFMS (*via* polling, *i.e.* periodic querying for new data, or by directly pushing any updates) once the calculator task is completed (successfully or with an error). This model is often imposed by necessity of dealing with computational scheduling software, which may not make intermediate results available to the WFMS for performance reasons. This also encourages splitting complex tasks (*e.g.* during geometry optimisation using energy gradients computed by finite differences) into smaller pieces (*e.g.* single point energy calculations of the displaced geometries) for optimal use of resources *via* task scheduling, with any decision making (*e.g.* evaluation against convergence criteria, and, if necessary, a next optimisation step) carried out at the workflow level.

**Fig. 2:**
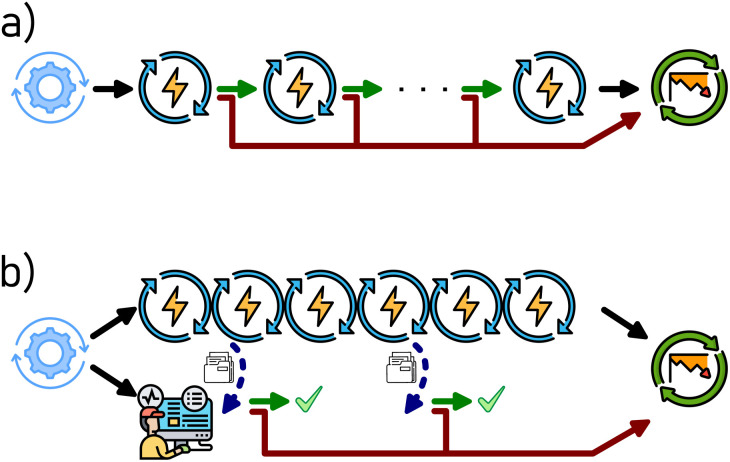
Example coin cell battery workflows, showing (a) a linear workflow with a decision making step after every charge/discharge cycle, and (b) a workflow with continuous cell cycling accompanied by a decision making *via* job monitoring.

The same approach could, in principle, be applied to experimental tasks. For instance, if we were to design a workflow for testing the degradation in the capacity of a coin cell battery, we may opt to split it into individual charge/discharge cycles, and evaluate the capacity of each completed cycle against the criteria at the workflow level. This is shown in Fig. 2(a), where the workflow consists of a pretreatment step (“formation cycles”), the main task which is split into its components (“charge/discharge cycles”), and finally any further steps (here a “safety discharge” to allow safe sample disposal). However, from an experimental point of view, this is often impractical, as the decision making process introduces an undefined delay between the individual cycles, which might be problematic in distributed systems with many parallel workflows. In particular, prolonged storage of the coin cell at a state of high charge or deep discharge may affect the cell performance,^41^ introducing an unacceptable degree of uncertainty into the workflow.

**Fig. 3:**
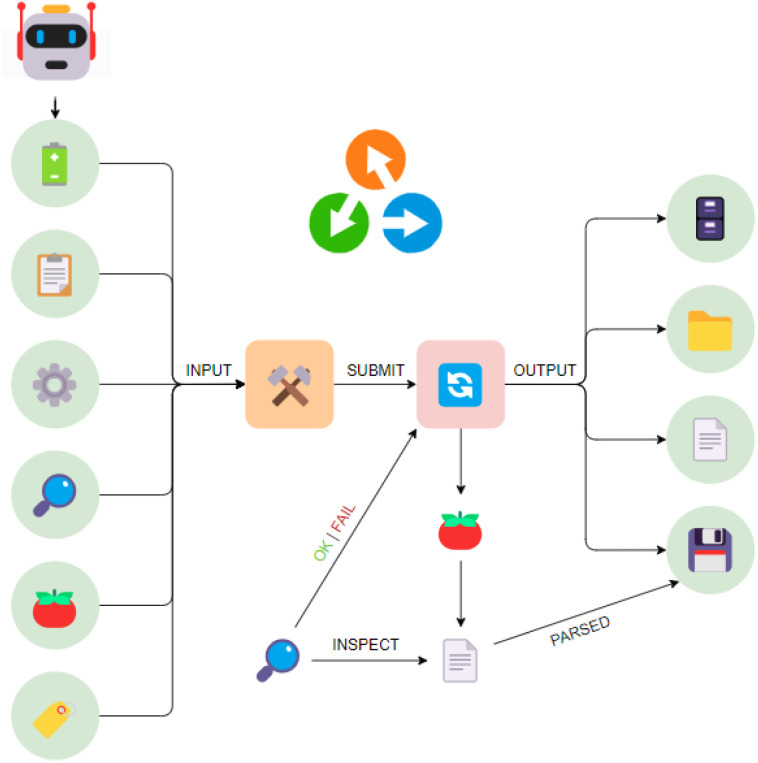
A stylised representation of typical provenance graphs of experiments submitted from AiiDA. Starting in the upper-right of the figure, the cell assembly robot output is first parsed into a digital twin of a battery sample, represented by the green Data node. This node, along with other Data nodes containing the protocol(s), the tomato settings, the job monitoring settings, the tomato code, and tags, are provided to AiiDA to construct a CyclingSequenceWorkChain node (orange). This CyclingSequenceWorkChain then submits each protocol in order, represented by the BatteryCyclerExperiment nodes. If requested, a job monitoring function (represented by the magnifying lens) will inspect, at a set interval, a snapshot of the experimental data produced by tomato, and terminate the experiment according to the specified criteria. Final results, regardless of termination by the job monitor, are parsed by AiiDA into an ArrayData node, assigned as an output of the BatteryCyclerExperiment, along with references to the remote working directory, the local working directory, and the retrieved raw data file. Example provenance graphs obtained from real data using AiiDA's verdi plot utility are shown in the ESI;† further information about AiiDA's provenance model is available online.§§See the “Provenance Implementation” entry in the topics section of AiiDA documentation, available at: https://aiida.readthedocs.io/projects/aiida-core/en/v2.2.0/topics/provenance/implementation.html

Instead, we introduce the concept of job monitoring, and its implementation in AiiDA as of version 2.2.0, schematically shown in Fig. 2(b). In this case, the main cell cycling task is submitted as a single task, programmed to perform the maximum desired number of cycles. In parallel to the submission of the main task, AiiDA can be instructed to perform job monitoring of that task. This is achieved by periodically polling the main task for data, which, based on a pre-defined criterion, allows the main task to continue or triggers its early termination. Of course, the measured data of the terminated tasks is retrieved by AiiDA, with the task completion status (including any decision by the job monitor) annotated accordingly, as shown in the provenance graph in Fig. 3 and S1.† Further steps in the workflow, such as the safety discharge step shown here, are not affected.

The latter approach has several advantages. The decision making of the monitoring job is intentionally kept simple, with the only options being to abort the main task or to do nothing (*i.e.* continue the task). However, the decision criteria can be of arbitrary complexity, and can depend on quantities computable only after the main task is started. For instance, a common decision point in battery testing is when the capacity of the investigated battery cell drops below 80% of the capacity obtained from the first cycle after pre-treatment (*i.e.* after “formation cycles”).^42,43^ The exact first cycle capacity is not known *a priori*. While this criterium can easily be implemented using a job monitoring (Fig. 2(b)), it would require modification of the linear workflow shown in Fig. 2(a), as the first cycle is treated differently from every successive cycle. Perhaps the biggest advantage is in saving valuable instrument time: defective or poorly performing battery cells can be detected automatically and their study curtailed without human intervention. In view of battery research labs, where parallel cycling of several hundreds of cells is becoming common, the instrument time saved by job monitoring quickly becomes appreciable. Alternatively, the feature can be used to detect “steady states” in heterogeneous catalysis^11^ and thus accelerate catalytic testing workflows.^32^

The disadvantages of such approach are minor, but have to be considered. First, periodic data snapshots have to be available to AiiDA; this feature is implemented within tomato, but other schedulers might not support it. Second, in its current implementation, the job monitoring is performed on the same server where AiiDA is running, placing additional load on the hardware and increasing network traffic by repeated transfer of data snapshots; these issues can be alleviated by reducing the polling frequency of the monitoring job and by checksumming of snapshots. Finally, the monitoring job may abort the main task at an arbitrary point in its execution, leading to a poorly defined state of the sample (*e.g.* a state of partial charge); to account for this, judicious planning of the overall workflow is required, in our example achieved by implementing a final safety discharge task which is always executed.

Full description of monitoring jobs is included in the AiiDA documentation, available online.¶¶See the “How to monitor (and prematurely stop) a calculation” entry in the How-To Guides section of AiiDA documentation, available at: https://aiida.readthedocs.io/projects/aiida-core/en/v2.2.0/howto/run_codes.html#how-to-monitor-and-prematurely-stop-a-calculation Currently any Python function matching a defined function signature can be used as the decision criterium; we hope to provide a domain-specific library of such functions in the future to enable reproducible testing. Note that all quantities known to AiiDA can also be accessed from within this Python function, including data obtained from previous or parallel steps and workflows.

### 2.4 Incorporating data from external sources

In most cases, labs undergoing automation and digitalisation will have “legacy” data, or will continue to generate data from external sources, *i.e.* out-of-band of the automation process that is being implemented. Such out-of-band data was also generated as part of the current work. However, by incorporating appropriate data parsing routines into our FAIR data parser yadg,^34^ we are able to ingest such out-of-band data into AiiDA and collate it under the appropriate digital twins. As shown in Fig. S2,† the provenance graph of fully in-band experiments can be mimicked in this way. As will be shown below, the ingested out-of-band data can be post-processed using the same tooling as the in-band data. Currently, the provenance graph for such out-of-band experiments may be incomplete. Solutions for automating and/or facilitating the reconstruction of the relevant metadata, which may be currently missing, will be explored in a future work, provided the required data is available.

## 3 Case study

In the following sections, we present the user interfaces developed for battery research and packaged in the AiiDAlab-Aurora plugin. Installation instructions are available in the ESI,† as well as in the project documentation.||||See the “Installation Guide” entry of the AiiDAlab-Aurora plugin, available at: https://aiidalab-aurora.readthedocs.io/en/latest/installation/index.html The sections of the case study are laid out following the chronology of a typical study. To better illustrate the above implementation, we have prepared several batches of coin cell batteries using the coin cell assembly robot, and studied the degradation in their discharge capacity as a function of upper cut-off voltage over the first 50 cycles. A video illustrating the interaction with the interfaces shown in the screenshots is available on the BIG-MAP app store, see the ESI† for details.

### 3.1 Materials

The coin cells are assembled into a CR2032 form factor using the coin-cell assembly robot, co-developed with Chemspeed Technologies, which is part of the Aurora platform. Batches of 32 cells can be routinely assembled. We use a 15 mm diameter graphite electrode as anode (1.2 mA h cm^−2^, Customcells), and a 14 mm diameter nickel-rich lithium nickel manganese cobalt oxide (NMC622) electrode as cathode (1.0 mA h cm^−2^, Customcells). As electrolyte, we use 100 μl of a 1 M solution of lithium hexafluorophosphate (LiPF_6_) in a mixture of ethylene carbonate (EC) and ethyl methyl carbonate (EMC) in a 3 : 7 ratio (Solvionic), respectively. The electrolyte is dispensed using a gravimetric dispensing unit (Chemspeed Technologies GDU-V) and also contains 2 wt% of vinyledene carbide (VC) to aid formation of a passivating solid-electrolyte interphase on the graphite electrode. Glass fiber separators (Whatman) are used to separate the electrodes; compatibility of the assembly robot with polyolefin separators (*e.g.* Cellgard) is being improved. Based on the cathode areal capacity and its electrode area, the corresponding target “recipe” capacity of each cell is 1.54 mA h.

The assembled cells are manually loaded into a set of 16-channel potentiostats (BioLogic MPG2). They have to be marked as available for use by tomato, by matching the cell name with the physical channel of the potentiostat, and marking the channels as ready.

### 3.2 Creation of digital twins

**Fig. 4:**
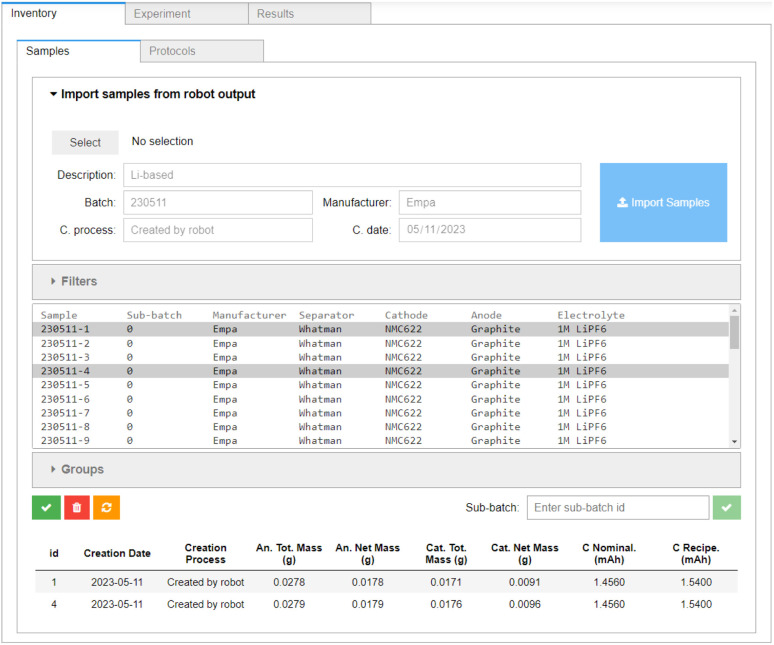
The *Inventory* → *Samples* component of the AiiDAlab-Aurora user interface, showing a sample creation widget for importing the output files from the cell assembly robot, the sample selector, and sample metadata. A sub-batch can be defined *via* multiple selection and the text field shown.

Once the cells are assembled, the output file of the cell assembly robot can be directly processed using the *Inventory* → *Samples* tab of the AiiDAlab-Aurora interface, shown in Fig. 4. The digital twins of the individual cells (*i.e.* samples) are prepared from this data, and, for now, stored within AiiDAlab. In the future, a direct connection to electronic lab notebooks is planned, which will allow for the complete sample history and other relevant information to be fetched, as necessary. They are pushed into AiiDA at the workflow submission stage, see below. As the assembly robot contains a balance, the masses of the anode and cathode in each individual cell are known, and the nominal cell capacity (denoted “C Nominal” in Fig. 4) can be recorded alongside the “recipe” capacity (1.54 mA h). In principle, this nominal cell capacity can be used to determine the charge and discharge rates, which may be especially important in cells with a large variation in electrode mass. However, in the current work, we use the “recipe” cell capacity to set the charge and discharge rates. The electrode masses are well behaved (with mean masses of 28.12(19) mg and 18.05(33) mg for the cathodes and anodes of the first batch (230511), and 28.05(27) mg and 17.86(37) mg for the cathodes and anodes of the second batch (231012), respectively). For an overview of the individual electrode masses of the assembled cells, see the ESI.†

### 3.3 Battery cycling protocols

The two batches of 32 coin cells have been tested according to the following workflow, with each step corresponding to a single protocol:

• Protective charge. The cell is charged up to 2.5 V at C/10, and then kept at a constant voltage of 2.5 V for 15 minutes, or until the charging current drops below C/20. This is followed by a cell relaxation (open circuit) for 6 hours.

• Formation cycles. Three charge–discharge cycles, with a charge to an upper cut-off voltage of 4.2 V at C/10, and a lower cut-off voltage of 2.5 V during discharge at D/10.

• Long-term cycling. Each batch of 32 cells is split into 4 sub-batches of 8 cells each, with different upper cut-off voltages. Each cell is cycled for up to 700 charge–discharge cycles, with an upper cut-off voltage of 4.2, 4.4, 4.6, or 4.8 V during charge at 1C, and a lower cut-off voltage of 2.5 V during discharge at 1D.

• Capacity monitoring. The long-term cycling of cells is stopped for any cell for which the capacity in discharge has dropped below 80% of the first cycle capacity (in discharge), in at least 3 consecutive cycles.

• Safety discharge. Discharge at 1D with a lower limit of 2.5 V.

**Fig. 5:**
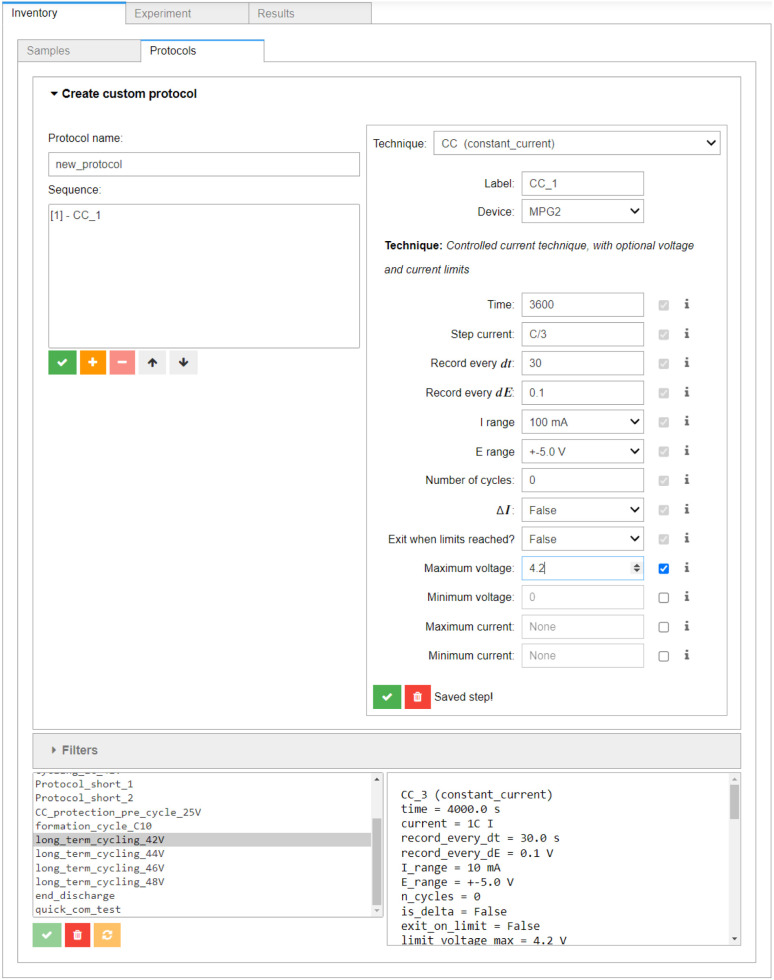
The *Inventory* → *Protocols* component of the AiiDAlab-Aurora user interface. The widget for building protocols out of electrochemical techniques, as well as the list of previously created protocols, are shown.

The above workflow is loosely based on the Base Cycling Protocol specified in BIG-MAP Deliverable 8.1.^44^ The individual protocols can be designed using the *Inventory* → *Protocols* section of the AiiDAlab interface (see Fig. 5), which is modelled after the protocol builder in BioLogic's EC-Lab software. Each protocol can be saved in AiiDAlab for later re-use, and can be submitted as an individual task, or as an AiiDA workflow, see below.

### 3.4 Reproducible workflows

**Fig. 6:**
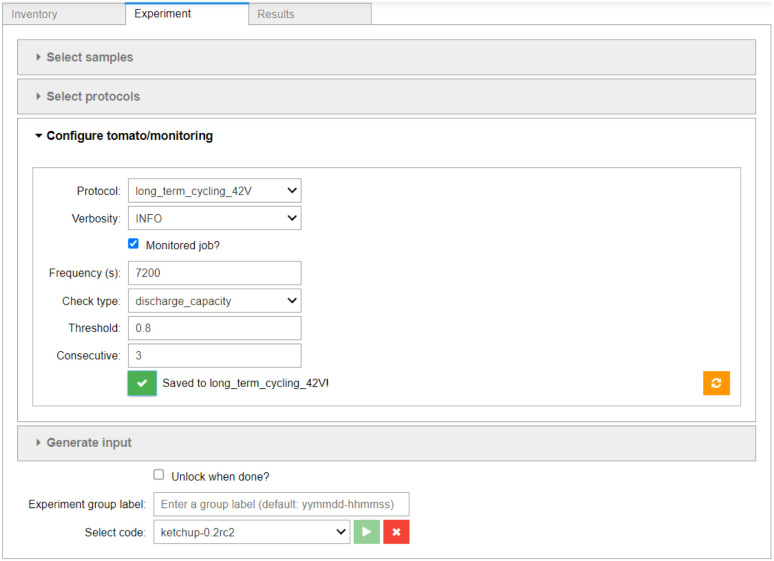
The *Experiment* component of the AiiDA-Aurora user interface. The sample and protocol selection widgets are collapsed, see the ESI† for their expanded versions. The widget for configuration of job monitoring is shown.

Having described our samples and defined our protocols, the *Experiment* component (shown in Fig. 6) can be used to assemble the experimental workflow. The *Select samples* widget (shown in Fig. S3†) allows for filtering and efficient selection of multiple samples in order to submit batches of experiments easily. The *Select protocols* widget (shown in Fig. S4†) allows for arranging individual protocols into a linear workflow. The configuration of the job monitoring is done on a per-protocol basis, using the interface shown in Fig. 6. Here, the user is asked to select the monitoring frequency, the monitoring function used (here: capacity in discharge), and supply any parameters required by the monitoring function (in this case the capacity threshold of 80% and number of consecutive cycles for which the condition has to be met).

Finally, the user is given the option to review the input and provide an arbitrary text label for this group of experiments, defaulting to the submission timestamp (shown in Fig. S5†). After selecting the appropriate “code” (*i.e.* an executable accessible by AiiDA on a local or networked computer, in this case the ketchup utility, which is part of tomato), the batch of experiments can be submitted. AiiDAlab then passes the specifications of the cells and protocols to AiiDA, which creates the appropriate nodes and digital twins, and manages the data retrieval without further action by the user.

For the purposes of this case study, one cell batch is submitted for testing using AiiDAlab (*i.e.* using the full software stack shown in Fig. 1), while a second batch is submitted manually, using an equivalent testing protocol implemented using the GCPL technique in EC-Lab software (version 11.48), representing a set of out-of-band data.

### 3.5 Accessing cycling results

**Fig. 7:**
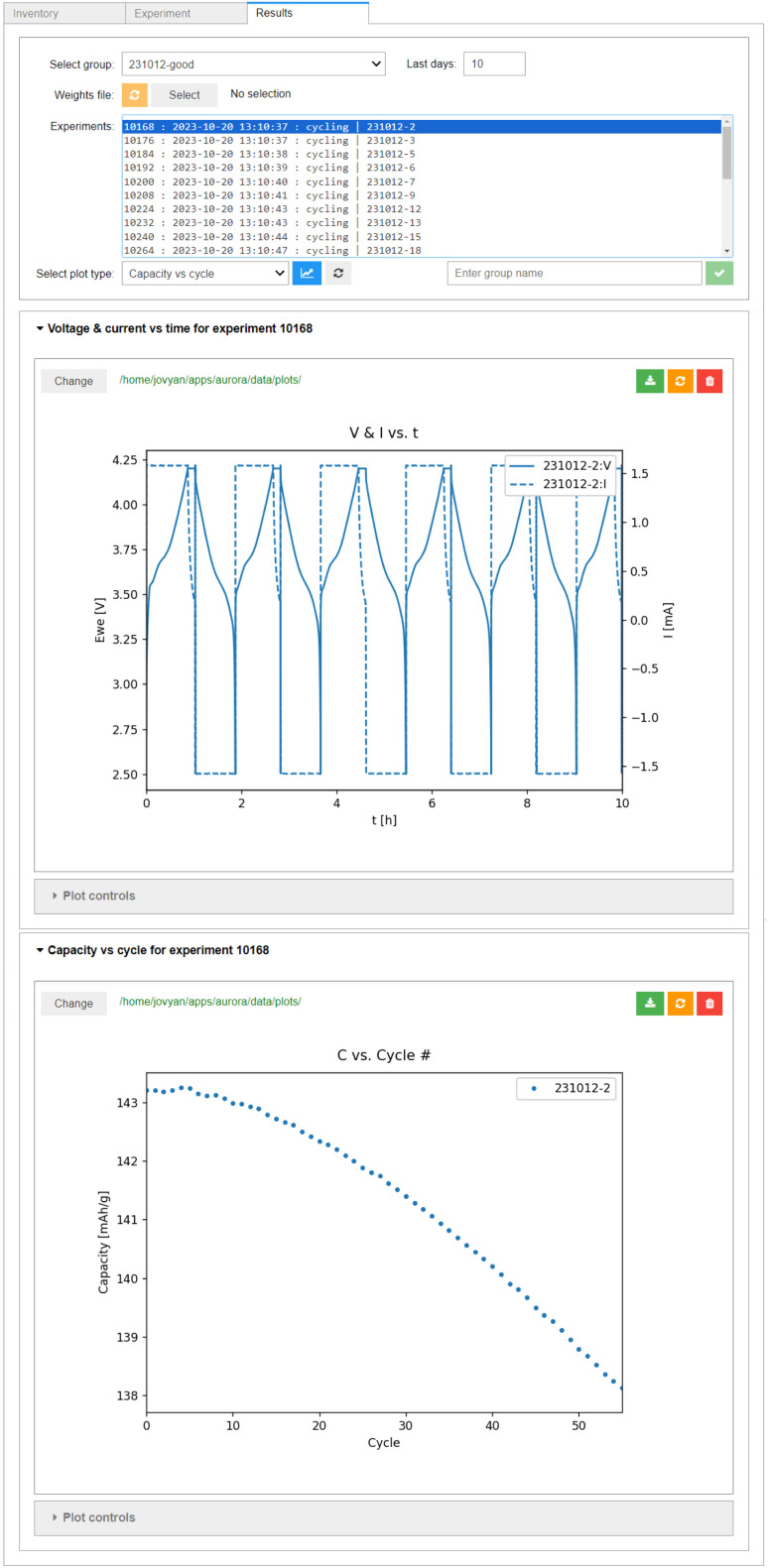
The *Results* interface, showing two plots of results of a single experiment. The upper panel shows the raw data (cell voltage *E*_WE_ and current *I* as a function of time). The lower panel shows a plot of processed data (capacity in discharge as a function of cycle number).

The results of the experiments are retrieved automatically by AiiDAlab, once the user enters the *Results* interface. Several pre-configured plots are available for analysis of single cells. As shown in the top panel of Fig. 7, all raw data is available (here, cell voltage and instantaneous current) as a function of time. However, an arbitrary amount of data post-processing can be implemented during the ingestion of the raw data archives into AiiDA. This is shown in the bottom panel of Fig. 7, where the derived data, in this case the cell capacity in discharge (calculated from an integral of the discharge current over time) is plotted as a function of cycle number (determined using changes in the sign of the supplied/drawn current).

**Fig. 8:**
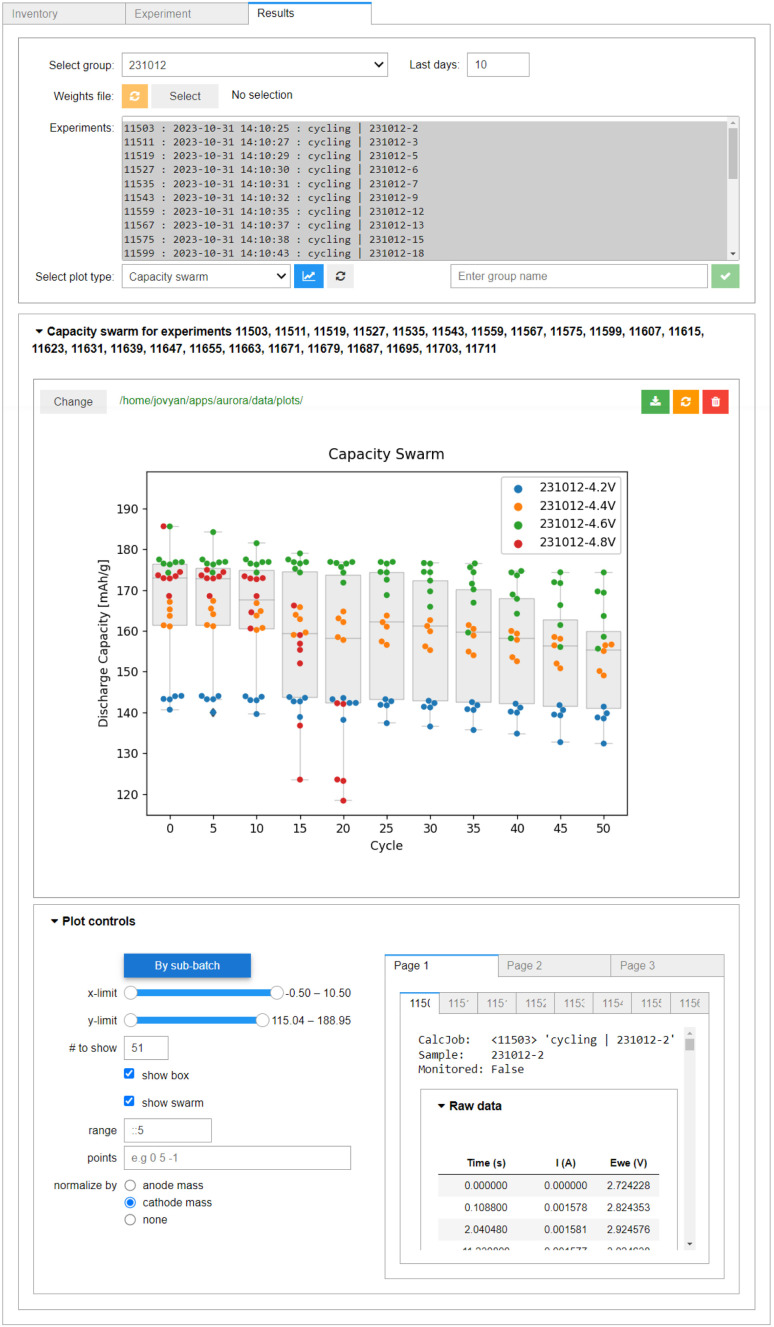
The multi-cell plotter within AiiDA-Aurora, built for analysing capacity retention as a function of cycle number. The box plots correspond to the summary statistics of the whole batch of cells (here, batch 231012). The colours of the swarm plots correspond to the four sub-batches within the batch (defined by their upper voltage limit: 4.2 V in blue, 4.4 V in orange, 4.6 V in green, 4.8 V in red). In the *Plot controls* panel, the axis limits can be adjusted, using Python-like syntax. The *y*-axis can be optionally normalized by electrode mass, the colouring of swarm plots by sub-batch can be toggled, and an overview of the raw data for each plotted sample is provided. Note that only 25 out of the 32 cells in batch 231012 are shown, see ESI (Fig. S6†) for details.

The data ingested into AiiDA is, by necessity, standardised. This means the whole experimental batches can be quickly screened for interesting results, for example using the box/swarm plot interface shown in Fig. 8. Here, the cell capacity in discharge normalised by the mass of the cathode active material is plotted as a function of the cycle number. In principle, any of the data “columns” attached to the digital twins can be plotted. The plotter also handles tasks terminated *via* job monitoring. Summary statistics of the overall selection are available *via* box plots, meaning the evolution of a batch of samples can be analysed quickly in a single plot. Individual cell data or sub-batches can be tagged using arbitrary metadata labels (here upper cut-off voltage) and visualised using swarm plots (shown as different colours). As we would expect, a higher cut-off voltage during cycling leads to a higher specific capacity, but also a much poorer capacity retention. In fact, the red swarm plot shows that all 8 of the cells cycled up to 4.8 V were terminated during the first 20 cycles *via* job monitoring, as their capacity in discharge dropped below 80% of the capacity during the first discharge. The *Plot controls* widget, shown in the lower panel of Fig. 8, allows for an interactive adjustment of the plot.

### 3.6 Results incorporating externally gathered data

**Fig. 9:**
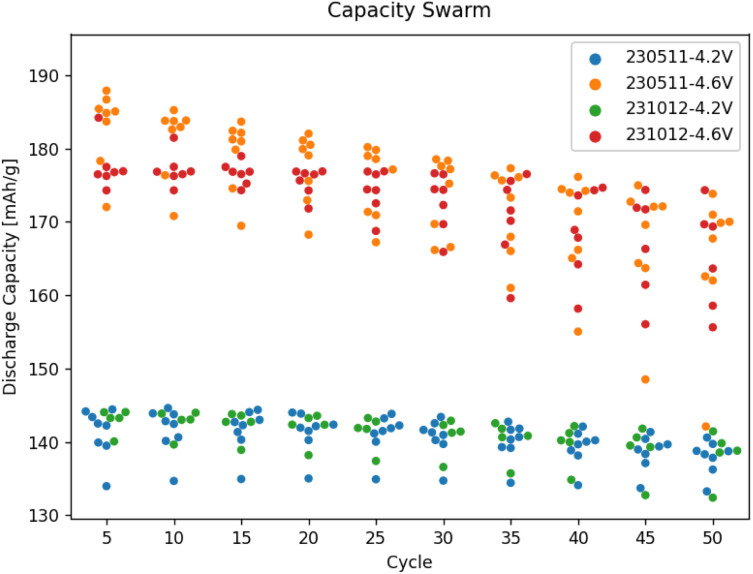
A comparison of the capacity degradation of selected cells in the two cell batches assembled using Aurora, with one batch (231012) tested in-band using AiiDAlab-Aurora, and the second batch (230511) tested out-of-band of AiiDA, using EC-Lab. Only two of the four sub-batches of cells shown, a plot of all retrieved data is available in the ESI, see Fig. S6.†

Finally, we illustrate the capability to plot data gathered externally along with data from the workflows submitted using AiiDAlab. Both batches shown in Fig. 9 have been assembled by the robot and tested using equivalent protocols. However, for one batch (231012), the data has been recorded in-band *via* tomato, while for the other batch (230511), it has been recorded out-of-band, using EC-Lab. Yet, upon standardisation of the out-of-band data using yadg and ingestion into AiiDA, the data can be plotted side-by-side using the AiiDAlab-Aurora interface. The swarm plots shown in Fig. 9 and S6† indicate that at least for the 4.2 V, 4.4 V, and 4.6 V sub-batches, there is no statistical difference between the two batches of cells.

## 4 Conclusions

We have successfully built a bridge between trust, provided by computational workflow management systems, and control, represented by experimental instrument automation tools. In this way, experiments can be controlled, scheduled, monitored, and analysed analogously to computational workflows, all while maintaining the chain of trust by recording provenance information.

The case study presented in our work focuses on battery cycling experiments. The whole battery cycling process, starting with a successful cell assembly by a purpose-built robot, including the necessary formation cycles and long-term cycling using off-the-shelf equipment, and ending with a discharge step allowing for a safe disposal, is tracked and collated using digital twins. Protocols as well as data are included in the provenance graph. We stress that while the case study focuses on battery testing experiments, the implementation is flexible enough to allow adaptation to other experimental workflows, such as in catalysis.

Additionally, we have demonstrated that the workflows can be reused, periodically monitored, and curtailed automatically based on arbitrary termination criteria – features with potentially large cost and time savings, both in computational and experimental research. Finally, we have developed and interfaced with parsers for external, legacy, or out-of-band data sources, aiding the transition from manual to automated labs by allowing ingestion of existing data, and its plotting along results obtained in-band.

## Data availability

All software developed as part of this work is made available using open-source licenses, and can be downloaded *via* the BIG-MAP app store, available at: https://big-map.github.io/big-map-registry/. The user interfaces shown above are contained in an AiiDAlab plugin, AiiDAlab-aurora, with screenshots obtained using version 0.11.2 of the plugin. The communication between AiiDA and the autonomous robotic battery materials research platform Aurora is handled by an AiiDA plugin, aiida-aurora, with version 0.4.4 used in this work. Additionally we used AiiDAlab version 23.3, AiiDA version 2.4.0, tomato version 0.2, and yadg version 4.2.4. The data for the two batches of cells presented in the case study is available in the ESI, see https://doi.org/10.5281/zenodo.10020712.

## Author contributions

Peter Kraus: conceptualization, data curation, investigation, methodology, software, validation, visualisation, writing – original draft, writing – review and editing. Edan Bainglass: data curation, formal analysis, investigation, methodology, software, validation, visualisation, writing – review and Editing. Francisco F. Ramirez: data curation, software, visualisation. Enea Svaluto-Ferro: data curation, formal analysis, investigation, visualisation. Loris Ercole: conceptualisation, methodology, software. Benjamin Kunz: investigation. Sebastiaan P. Huber: software. Nukorn Plainpan: software. Nicola Marzari: conceptualization, funding acquisition, resources, supervision, writing – review and editing. Corsin Battaglia: conceptualization, formal analysis, funding acquisition, methodology, project administration, resources, supervision, writing – review and editing. Giovanni Pizzi: conceptualization, funding acquisition, methodology, project administration, resources, software, supervision, writing – review and editing.

## Conflicts of interest

There are no conflicts to declare.

## Supplementary Material

TA-012-D3TA06889G-s001
