# Supplementary material for: A bridge between trust and control: computational workflows meet automated battery cycling
Source: J Mater Chem A Mater. 2024 Apr 3;12(18):10773–83. doi: 10.1039/d3ta06889g (PMC11077506; doi:10.1039/d3ta06889g)
Supplement: TA-012-D3TA06889G-s001 [file TA-012-D3TA06889G-s001.pdf]

Electronic Supplementary Material (ESI) for Journal of Materials Chemistry A.
This journal is © The Royal Society of Chemistry 2024

A bridge between trust and control: Computational workflows meet automated battery cycling

P. Kraus, E. Bainglass, F. F. Ramirez, E. Svaluto-Ferro, L. Ercole, B. Kunz, S. P. Huber, N. Plainpan, N. Marzari, C. Battaglia, G. Pizzi

E-mail: peter.kraus@tu-berlin.de

Version 0.1 – 7th of November 2023

Supporting information

This document is part of the Electronic Supporting Information archive for the above manuscript, available on Zenodo under DOI: 10.5281/zenodo.10020712. This document contains the following:

- A set of representative provenance graphs, automatically generated using `AiiDA`
- A set of supplementary screenshots of the *Experiment* component of the `AiiDAlab-Aurora` user interface
- A figure containing the cell cycling results for all cells in the two studied batches

Further information and data is available on the above DOI. Please contact Peter Kraus for any queries about the ESI.

Automated AiiDA provenance graphs

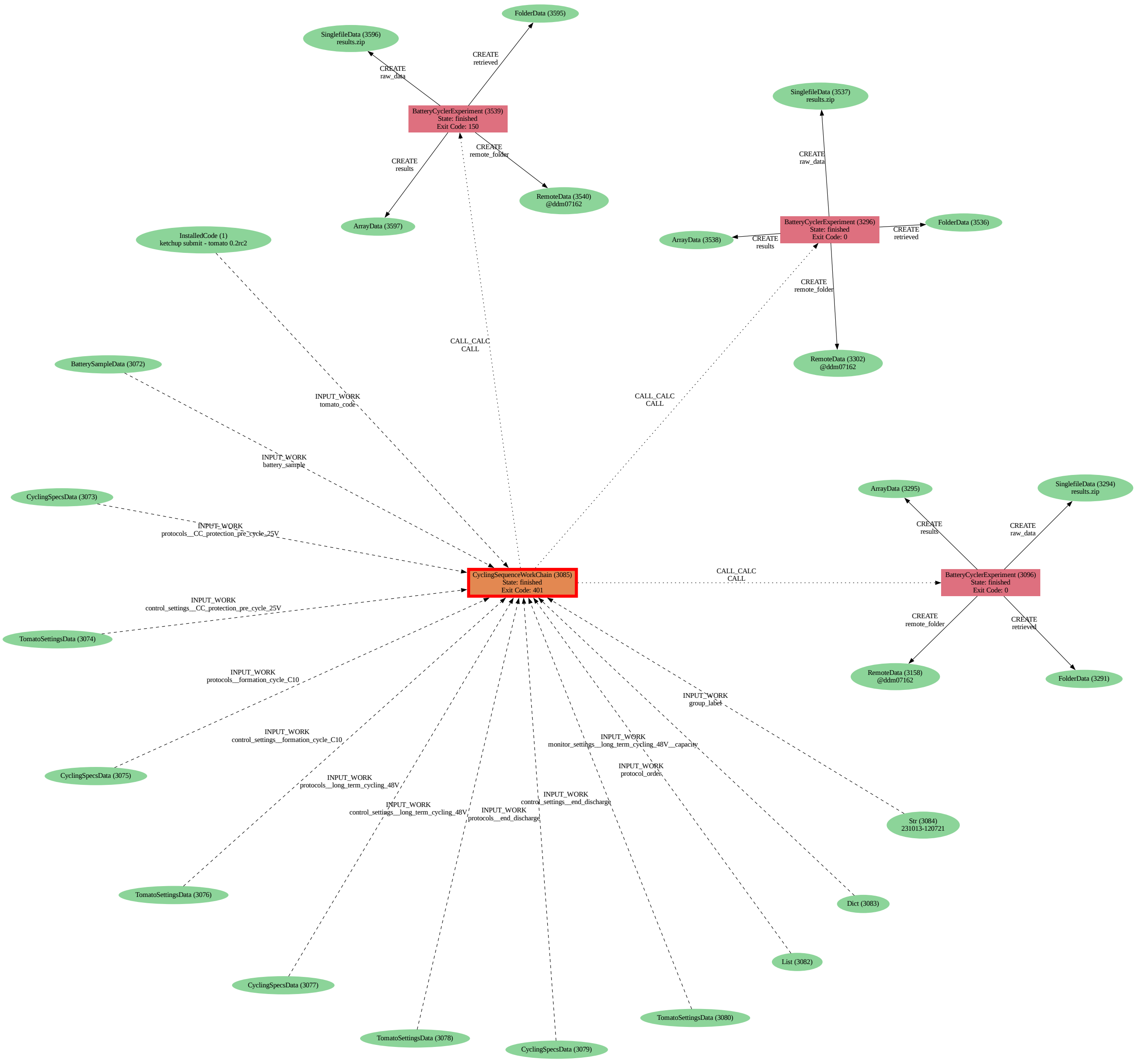

Figure S1: An automatically generated provenance graph for an in-band battery cycling workflow. The central `CyclingSequenceWorkChain` node (orange) corresponds to the overall workflow. It is linked to three `BatteryCyclerExperiment` nodes (red), corresponding to the Protective charge, Formation cycles, and Long-term cycling protocols of the workflow. Note that the Exit Code for the Long-term cycling (upper `BatteryCyclerExperiment` node) is 150, denoting that the task was aborted via job monitoring; the other two `BatteryCyclerExperiments` have an Exit Code of 0, denoting successful completion. The green nodes correspond to the various `Data` nodes, required to assemble the `CyclingSequenceWorkchain`, and retrieve the raw data from the remote host running `tomato`.

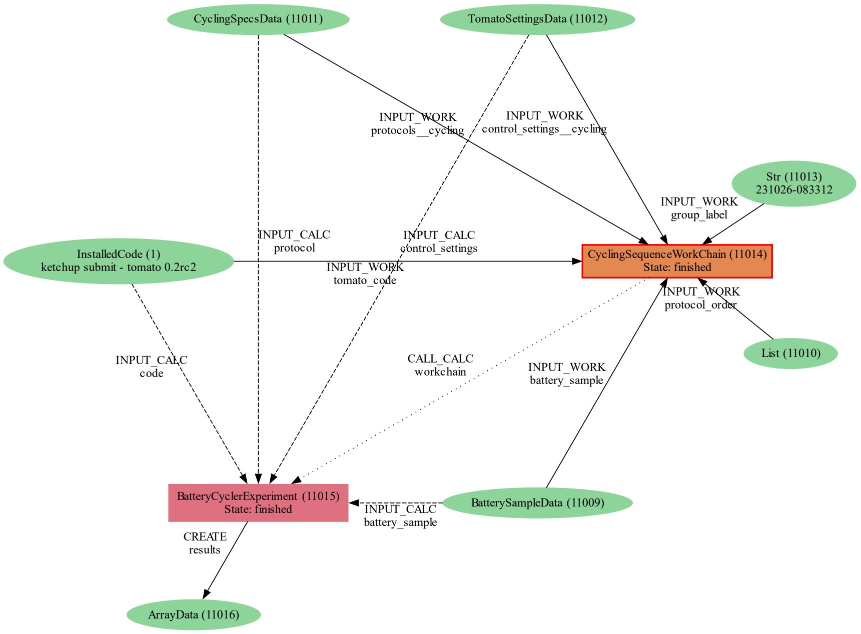

Figure S2: An automatically generated provenance graph for an out-of-band battery cycling workflow. At the centre is a simple `WorkChain` node (orange), linked to a single `CalcJob` node (cf. Fig. S1). However, provided the `Data` nodes used to construct the `WorkChain` contain the relevant metadata information and instructions, `AiiDA` is able to process the out-of-band data into the same internal format as that used for in-band data.

Supplementary `AiiDAlab-Aurora` screenshots

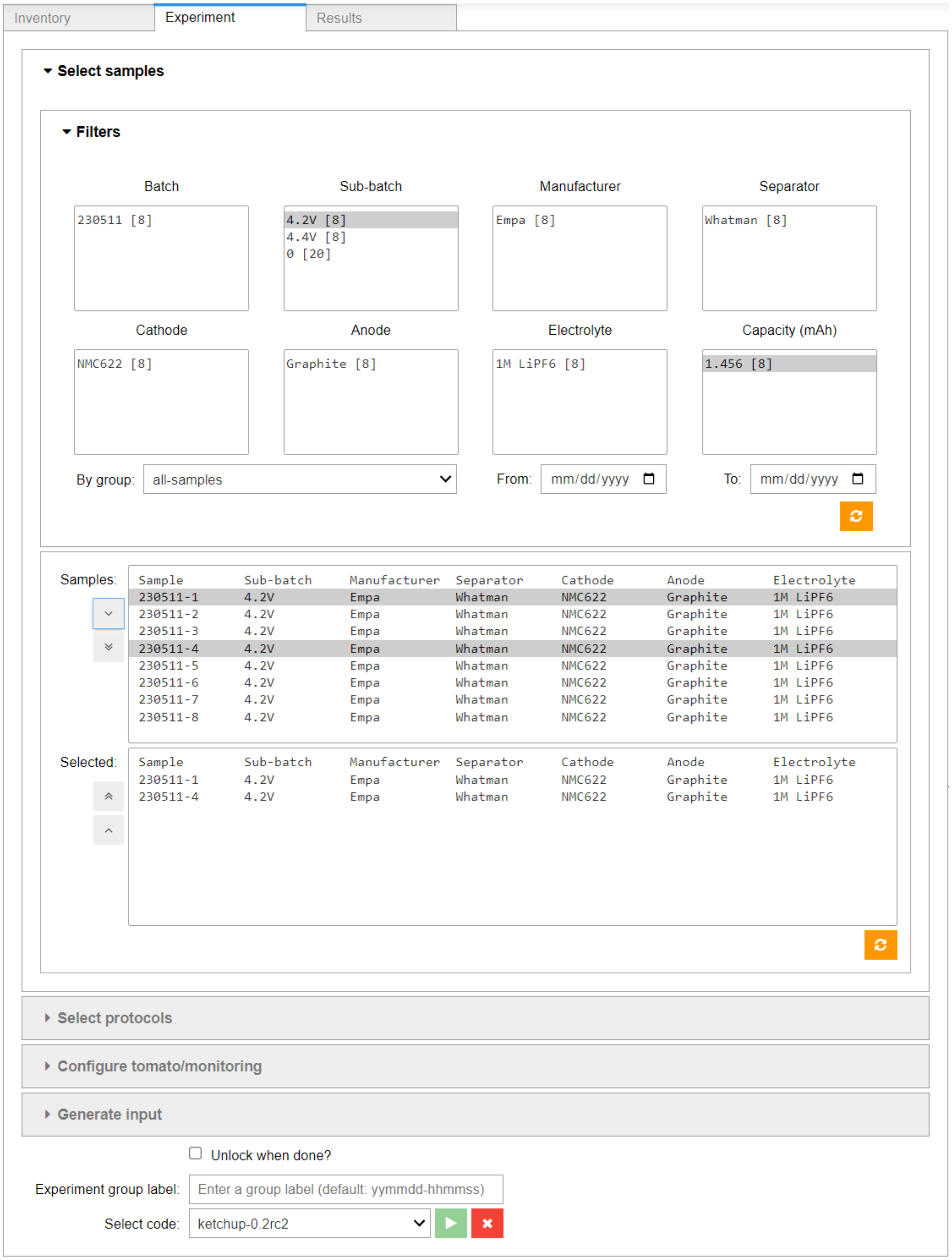

Figure S3: The *Select samples* section of the *Experiment* component of the `AiiDA-Aurora` user interface. This widget allows for filtering and selection of samples stored in the *Inventory*. The samples listed in the `Selected` section will be part of the workflow.

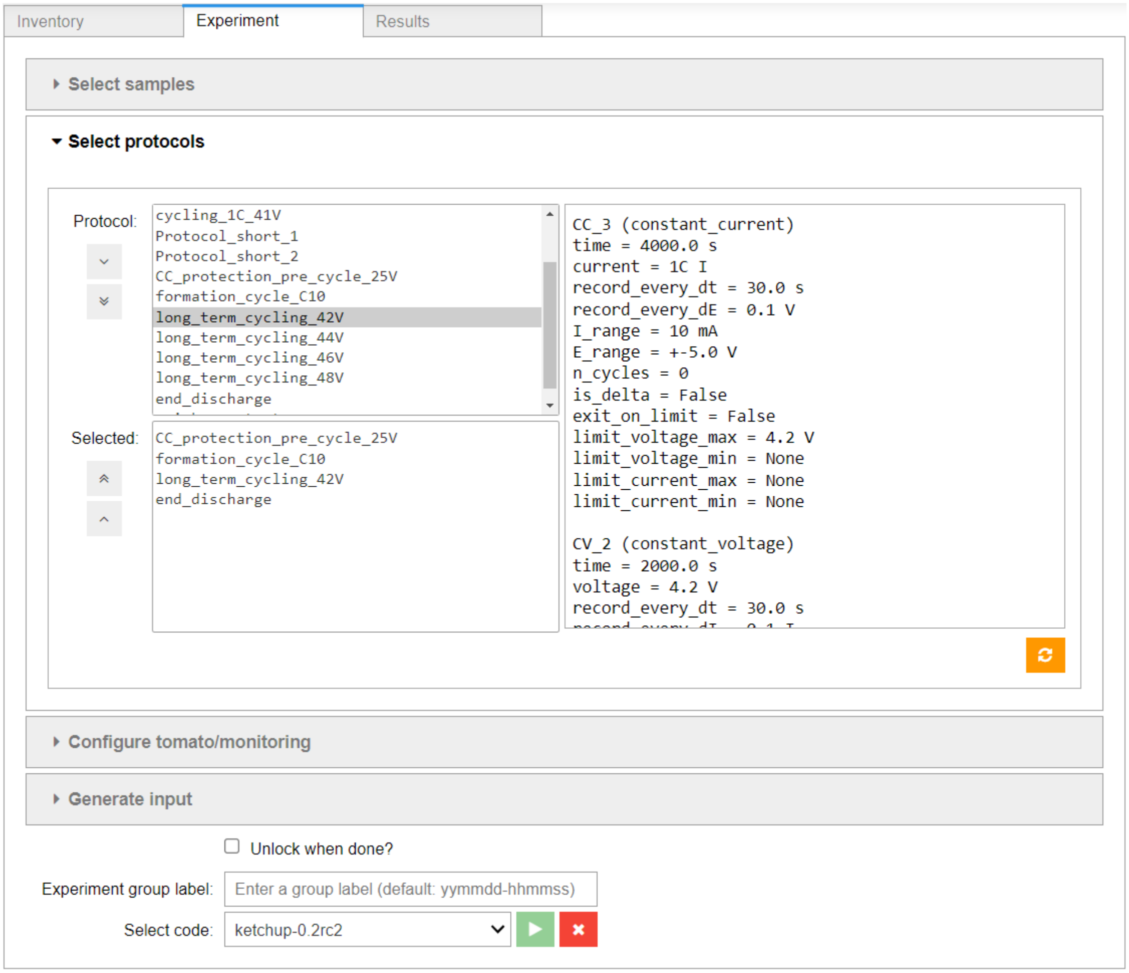

Figure S4: The *Select protocols* section of the *Experiment* component of the `AiiDA-Aurora` user interface. This widget allows for selection of protocols from the *Inventory*. The protocols will be executed in the order as listed in the `Selected` section.

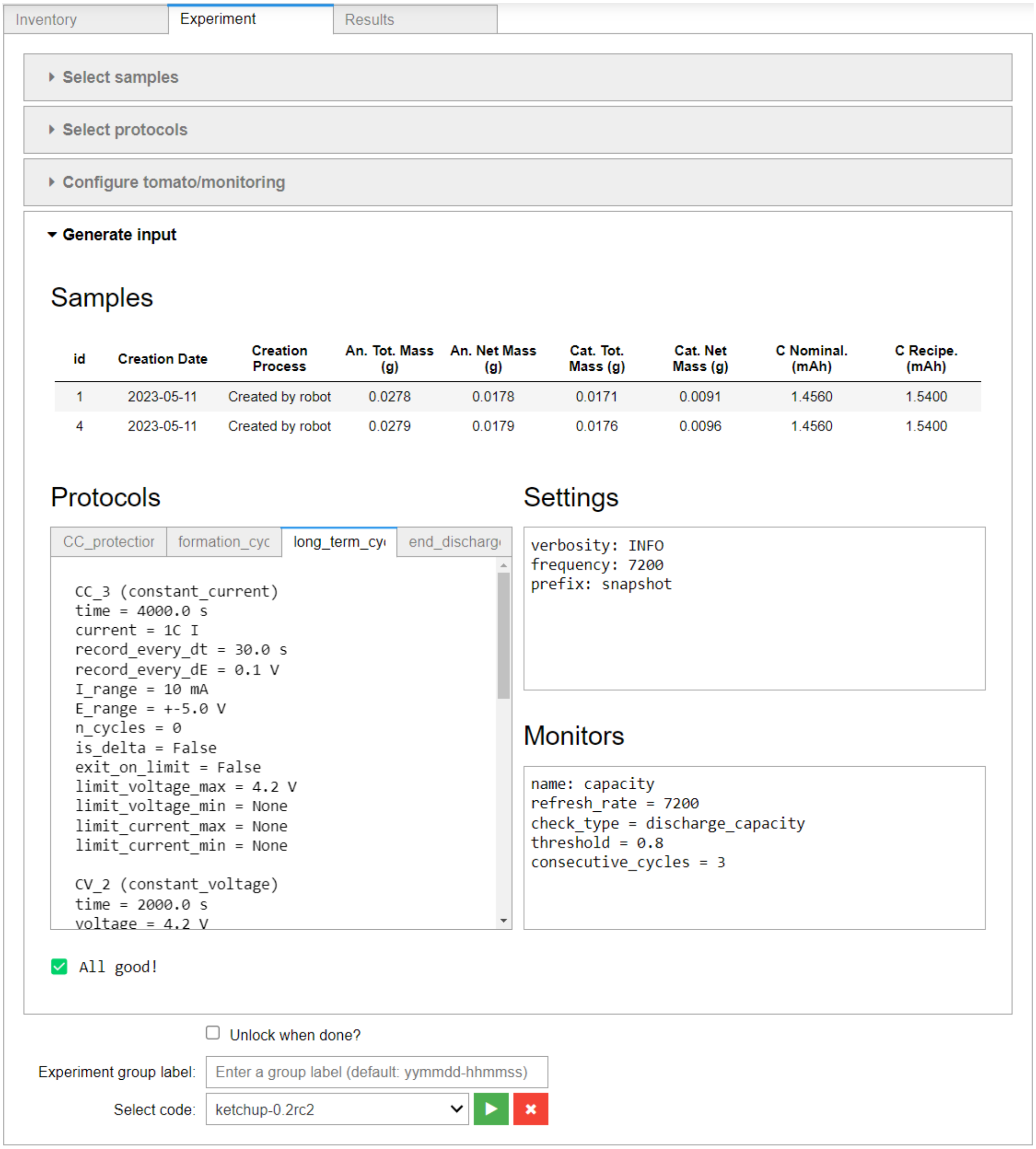

Figure S5: The *Generate input* section of the *Experiment* component of the `AiiDA-Aurora` user interface. This widget allows the user to review the selected samples as well as protocols and their monitoring settings prior to submission via `AiiDA`.

Complete cell cycling results

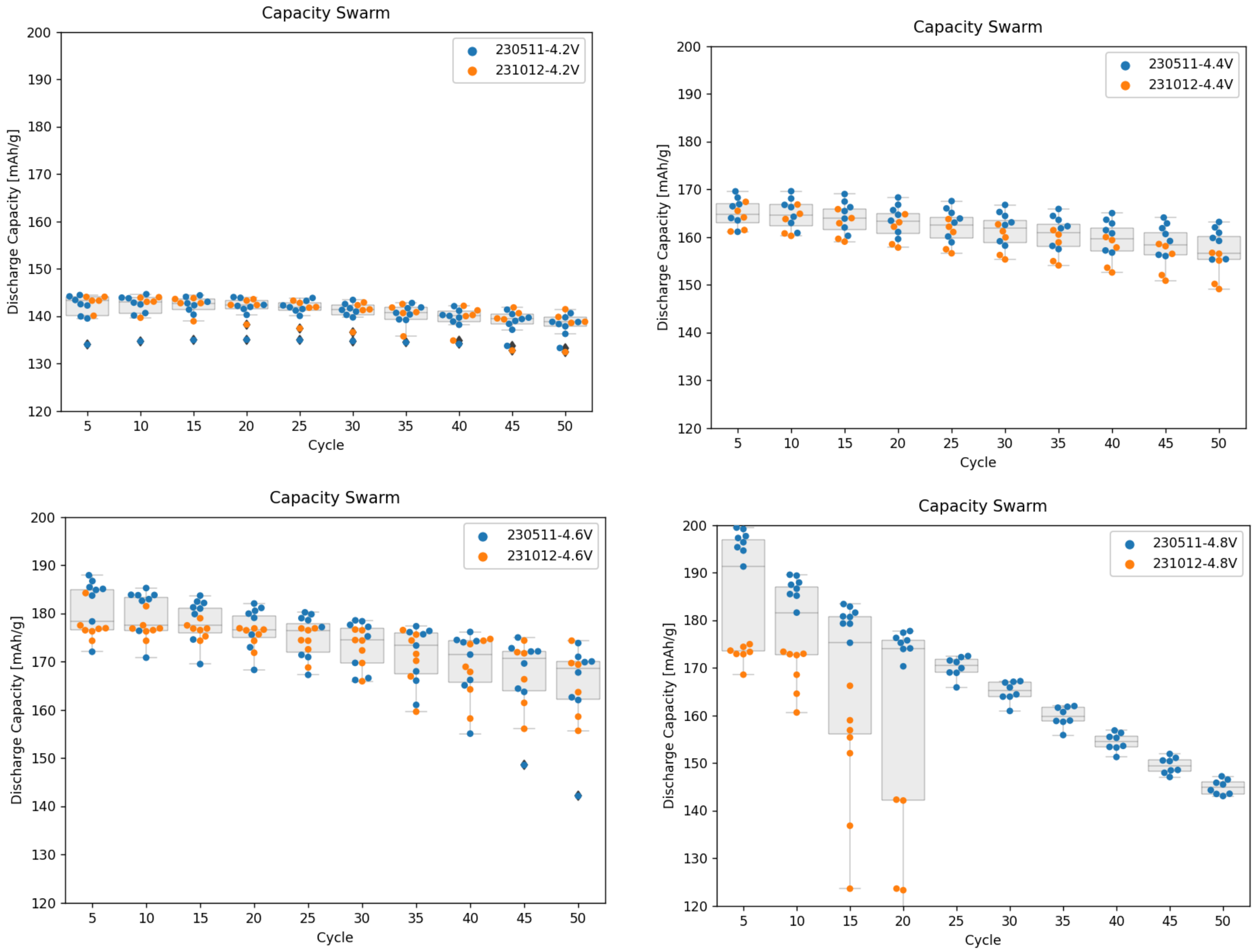

Figure S6: A comparison of the capacity degradation of all cells in the two studied cell batches. The colour of the swarm plots denotes batch of the cells. The data from the 230511 batch (blue) represents out-of-band data, as it has been imported into `AiiDA` from EC-Lab. The data from the 231012 batch (orange) has been gathered in-band, using an `AiiDA` workflow. The box plots show the statistics for all cells plotted. The behaviour of the cells cycled up to 4.2 V (upper left), 4.4 V (upper right), as well as 4.6 V (lower right) is consistent between the two batches. However, the cells cycled up to 4.8 V start at a vastly different capacity (∼175 mAh/g for the 231012 batch, vs ∼195 mAh/g for 230511), and their capacity degradation also seems to occur at different rates, with all cells in the 231012 batch (orange) stopped by the job monitor within 20 cycles. Note that for the batch 231012 (orange), only 25 out of the 32 assembled cells are shown, as 7 cells have failed before the first cycle, due to an assembly failure or a software error.
